# Effect of Atmospheric Cold Plasma Treatments on Reduction of *Alternaria* Toxins Content in Wheat Flour

**DOI:** 10.3390/toxins11120704

**Published:** 2019-12-03

**Authors:** Elizabet Janić Hajnal, Milan Vukić, Lato Pezo, Dejan Orčić, Nevena Puač, Nikola Škoro, Ardea Milidrag, Dragana Šoronja Simović

**Affiliations:** 1 Research Center for Technology of Plant Based Food Products, Institute of Food Technology, University of Novi Sad, 21000 Novi Sad, Serbia; 2 Department of Carbohydrate Food Engineering, Faculty of Technology Novi Sad, University of Novi Sad, 21000 Novi Sad, Serbia; milan.vukic@tfzv.ues.rs.ba (M.V.); dragana@tf.uns.ac.rs (D.Š.S.); 3 Department of Food Technology, Faculty of Technology Zvornik, University of East Sarajevo, 75400 Zvornik, Bosnia and Herzegovina; 4 Institute of General and Physical Chemistry, University of Belgrade, 11000 Belgrade, Serbia; latopezo@gmail.com; 5 Department of Chemistry, Biochemistry and Environmental Protection, Faculty of Sciences, University of Novi Sad, 21000 Novi Sad, Serbia; dejan.orcic@dh.uns.ac.rs; 6 Laboratory for Gaseous Electronics, Institute of Physics, University of Belgrade, 11080 Belgrade, Serbia; nevena@ipb.ac.rs (N.P.); nskoro@ipb.ac.rs (N.Š.); 7 Chair of general physiology and biophysics, Faculty of Biology, University of Belgrade, 11000 Belgrade, Serbia; ardea304@gmail.com

**Keywords:** wheat flour, atmospheric cold plasma, alternariol, alternariol monomethyl ether, tentoxin, LC-MS/MS, mathematical modelling

## Abstract

Beside *Fusarium* toxins, *Alternaria* toxins are among the most commonly found mycotoxins in wheat and wheat products. Currently, investigations of possibilities of reduction of *Alternaria* toxins in the wheat-processing chain are limited. Therefore, the aim of this study was to explore the potency of cold atmospheric plasma treatments, as a new non-thermal approach, for reduction of alternariol (AOH), alternariol monomethyl ether (AME) and tentoxin (TEN) content in spiked white wheat flour samples. Samples were treated with plasma generated in the air during 30 s to 180 s, with an increment step of 30 s, and at four varying distances from the cold plasma source (6 mm, 21 mm, 36 mm and 51 mm). The reduction of the *Alternaria* toxins content in samples after treatment was monitored by high performance liquid chromatography coupled to tandem mass spectrometry (LC-MS/MS). The maximum reduction of the examined *Alternaria* toxins was obtained by treatment performed at 6 mm distance from the plasma source, lasting 180 s, resulting in reductions of 60.6%, 73.8% and 54.5% for AOH, AME and TEN, respectively. According to the obtained experimental results, five empirical models in the form of the second-order polynomials were developed for the prediction of AOH, AME and TEN reduction, as well as the temperature and the moisture content of the wheat flour, that gave a good fit to experimental data and were able to predict the response variables successfully. The developed second-order polynomial models showed high coefficients of determination for prediction of experimental results (between 0.918 and 0.961).

## 1. Introduction

Wheat (*Triticum aestivum* L.) is one of the most important food crops, being among the ten most produced commodities [1,2]. The global quality of common wheat and wheat-based products are interrelated to various features such as protein content, protein quality, and presence of contaminants that affect the safety, generally associated with the absence of toxigenic molds and their secondary metabolites, mycotoxins [3]. Fungi of the genus *Alternaria* (about 250 species), can grow at low temperature. Frequency and ability of this genus to produce a wide range of toxins is a significant and present threat to the health of humans and animals. Due to their prevalence in various foods and cumulative effect [4], mutagenic, teratogenic and possibly carcinogenic effects of *Alternaria* toxins are possible. According to the European Food Safety Authority (EFSA), major risk factors for *Alternaria* toxin dietary exposure are cereals and cereal-based products [5]. The genus of *Alternaria* is commonly present in cereals, resulting in a high possibility of *Alternaria* toxin incidence and increased risk in affected wheat [6]. *Alternaria* toxins most frequently present on wheat include alternariol (AOH), alternariol monomethylether (AME), tenuazonic acid (TeA), tentoxin (TEN) and altertoxins (ATXs) [7]. In recent years, many studies showed that the prevalence of *Alternaria* toxins in wheat from Europe, Africa, Asia, Australia and North and South Americas should not be underestimated [8,9,10,11,12,13]. Prevention of contamination by *Alternaria* fungi is the most sensible and cost-saving approach to reducing the risks accompanied with the presence of *Alternaria* toxins. Still, this approach is not always possible with current agronomic and storage practices, when the environmental conditions favor the growth of toxigenic fungi [8].

Investigations regarding the fate of *Alternaria* toxins during wheat kernel and wheat flour processing are limited. Only a handful of studies refer to the investigation of the fate of AOH, AME and TeA during wheat cleaning, wheat milling and bread-making [14,15]. The abovementioned studies show that wheat cleaning reduces the content of AOH, AME and TeA through the removal of impurities, whereas wheat milling performs only toxin distribution into wheat flour mill streams, and the bread-making procedure only cause a reduction in AOH content. Further, the extrusion process provides possibilities for reduction of *Alternaria* toxins. In the investigation of the potential of simple pilot single screw extruder for reduction of AOH, AME and TeA in flour of whole wheat, the highest reduction of AOH (87.9%), AME (94.5%) and TeA (65.6%) was achieved when high raw material moisture (w = 24 g 100 g^−1^), high feeding rate (q = 25 kg h^−1^) and medium screw speed (v = 390 rpm) were applied [16].

Recently, application of cold atmospheric plasma got much attention as a novel non-thermal technology for the food industry [17,18,19]. Some of the advantages of this technology are high efficiency with short treatment time, lack of residues, a low or positive overall impact on the quality of treated food products, and reduced costs when using ambient air as the working gas [20]. Not long ago, the research focus of the plasma treatment was on its effects on pathogenic bacteria [21,22]. Lately, much attention has been paid to studies related to plasma effects on chemical and biological compounds which showed promising results [23,24,25]. Degradation of mycotoxins is being studied worldwide with different types of plasma sources. Cold atmospheric plasma could possibly overcome the greatest disadvantages of classical techniques and provide a sustainable solution for mycotoxins detoxification [26,27,28,29]. Cold plasma is a weakly ionized, quasi-neutral gas with rich chemistry containing a wide variety of highly reactive species and ultraviolet radiation that are shown to be efficient in degradation of mycotoxins. The formation of reactive species in the discharge takes place through complex reaction mechanisms which depend on several factors like type of power supply, frequency, gas used, geometry of the electrode system etc. [30,31,32]. The choice of the most efficient plasma source is at large extent governed also by the type of application. In order to get the desired plasma chemistry for destruction of toxins it is necessary to characterize in detail and optimize the plasma source.

Cold atmospheric plasma in air generates a favorable combination of ions, short- and long-lived reactive oxygen species (ROS) and reactive nitrogen species (RNS) that include OH•, O•, NO, O_3_, H_2_O_2_, NO_2_, etc. [33,34,35,36]. By varying the plasma source geometry and type of applied voltage signal discharge can be tuned to produce ozone as one of the prevailing reactive specie which degrades, to a certain degree, mycotoxins on its own [37,38]. However, in addition to ozone alone, cold plasma in air leads to the formation of radicals, ROS, RNS and ultraviolet light, culminating in greater mycotoxin degradation efficacy under shorter exposure times than ozone alone. Hence, the importance of other reactive species produced in plasma should not be overlooked [39].

With cold plasma technology, it could possibly be feasible to degrade mycotoxins effectively. Treatment would be more sustainable requiring smaller energy inputs and investment compared to classical techniques. To be applicable to treat wheat flour, cold plasma treatments need to be able to treat the bulk quantities of wheat flour. Multiple intrinsic and extrinsic parameters of wheat flour matrix, including its powdered form, possibly play a role in the effectiveness of cold plasma treatment. One of the plasma sources that can meet these criteria is surface dielectric barrier discharge (SDBD). Technology allows treating the samples at atmospheric pressure with air as the working medium, thereby reducing equipment cost and offering the great advantage of high adaptability and scaling up.

Previous studies indicate that cold atmospheric plasma is a promising technology for degrading of mycotoxins [27,28,40,41]. However, none of these studies included *Alternaria* toxins in wheat flour matrix. Thus, the aim of the present study was to investigate the effects of atmospheric SDBD cold plasma treatments on *Alternaria* toxin (AOH, AME and TEN) reduction in wheat flour. The experiments were carried out by using SDBD reactor to excite strongly oxidizing cold air plasma above the samples. To process the data obtained, the second order polynomial (SOP) models were used for mathematical modelling. Pattern recognition technique (Principal Component Analysis – PCA) was applied to the experimental data (used as descriptors) to characterize and differentiate among the observed samples. The optimum processing conditions were determined by standard score (SS) evaluation.

## 2. Results and Discussion

### 2.1. Optical Emission Spectroscopy and Electrical Characterization of Plasma Source

**Figure 1:**
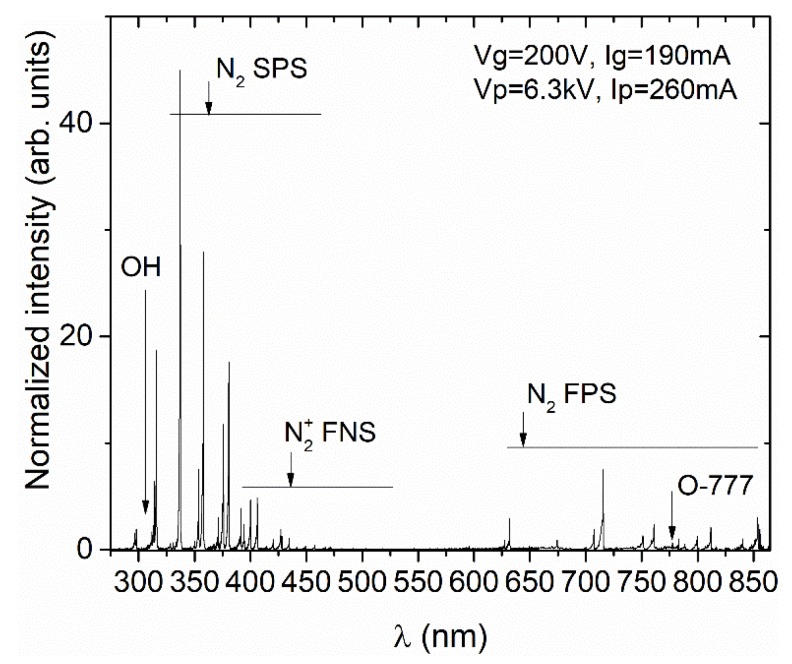
The optical emission spectra of the surface dielectric barrier discharge (SDBD) in atmospheric-pressure air.

Species is generally identified as the main active species responsible for the effects of cold plasma, among which is degradation of chemical and biochemical compounds are atomic oxygen, ozone (O_3_), hydrogen peroxides (H_2_O_2_), hydroxyl radicals (•OH), as well as peroxynitrites and nitrogen oxides [23,24,41]. Some of the species, such as •OH, NO, O, after excitation emit in near UV-visual range and this emission is easily recordable by the optical emission spectroscopy (OES) technique. An emission spectra of the discharge in air was recorded in a wide range of wavelengths from 270 nm to 860 nm by using the optical emission spectroscopy (OES) technique. Emission measurements were taken within the first few seconds after the plasma ignition through a quartz window inserted on one side of the plastic box. The spatial angle of the recording included the total volume of the discharge ignited at the lower side of the electrode as well as the volume directly under the electrode plate. The spectrum of the discharge (in arbitrary units) obtained in absence of flour samples is presented in Figure 1 and it gives us qualitative information regarding the chemical species present in the plasma discharge. The most intense lines recorded belong to the N_2_ Second Positive System band, as expected for the atmospheric air plasma. Relatively lower intensities of peaks associated with atomic oxygen (triplet at 777 nm) are due to involvement of O in creation of O_3_, and quenching by N_2_ and O_2_ molecules [42]. Since the plasma operates in ambient air with certain humidity, OH radicals are produced mainly through direct H_2_O dissociation. Thus, lines of the OH (A-X) band coming from excited OH radicals are also observed in the recorded spectrum with low emission intensity. Hence, the existence of these reactive species can be confirmed by OES characterization of the SDBD unit, thus showing the application feasibility to detoxicate samples with mycotoxins in general.

**Figure 2:**
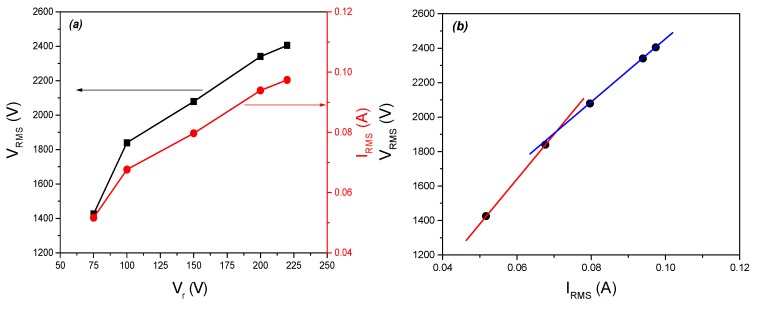
(**a**) Dependence of V_RMS_ and I_RMS_ values on the voltage values set by the variable transformer; (**b**) Voltage-current characteristics of the SDBD plasma system. Red and blue lines are guides for the eye for easier detection for impedance change. V_RMS—_root-mean-square value of voltage; I_RMS—_root-mean-square value of current; V_r—_voltage on variable transformer.

The high-voltage (HV) given by the transformer and the current were measured at the point of electrical circuit close to the powered electrode. The dependence of root-mean-square (RMS) values of voltage and current on the variable transformer (variac) voltage are shown in Figure 2a. As expected, for an increase in the variac voltage, there is an increase in both HV and current at the electrode. The maximal RMS values for voltage and current reached with this system were 2400 V and 0.1 A, respectively.

The voltage-current (V-I) characteristics of the system is shown in Figure 2b. At lower voltages immediately after the ignition, plasma does not cover the whole surface of the electrode, but with the increase in the applied voltage, plasma spreads and covers the whole surface uniformly. Spreading of the active plasma surface causes changes in the system impedance. This can be observed as a change from red to blue line in Figure 2b. For higher voltages, the impedance is slightly lower, which indicates larger electrode surface covered by homogenous plasma. Generally, when plasma is ignited the total impedance of the system is changed. The impedance change towards a lower value can be seen through the effective circuit of the system represented as parallel connection of two complex impedances. One impedance is due to the ignited plasma, which itself represents a complex RLC circuit, and the other one is the impedance of the electrode system. The impedance of the electrode system is due to the capacitance of the electrode system and, to some small extent, due to the resistance of the cables and connectors. Hence, in large area discharges at atmospheric pressure, the changes in the total impedance indicate the area covered by the discharge. Therefore, we have chosen 200 V as an operating voltage for the flour treatment since the plasma impedance (i.e., total impedance) is constant in this range of voltage-current parameters, since the whole electrode area is covered homogenously. Also, we avoided the highest achieved variac value because of the danger of temperature increase, as well as the change into arcing.

### 2.2. Reduction of Alternaria Toxins by Atmospheric Cold Plasma Treatments

Among the most commonly found *Alternaria* toxins in wheat [7], AOH, AME, and TEN, were chosen for the focus of this study. The results obtained by the analysis of *Alternaria* toxins in treated samples suggest that there is a realistic possibility of a significant reduction of toxins by cold atmospheric plasma treatment. The experimental results are presented in Table 1. The content of toxins was reduced even after only 30 s of treatment; the reduction ranged from 2.4% to 44.6%. The reduction rate was dependent on the toxin and sample distance from the plasma. The greatest reduction for all toxins was achieved when samples were placed 6 mm from plasma discharge, with AME showing the greatest instability. Similar behaviour of other toxins is noticeable from Table 1. Thus, it can be presumed that, in addition to duration and sample distance from the electrode, structural features of toxins affect the treatment efficiency.

For all treatment times, a similar reduction ratio between studied toxins is noticeable. AME was the most susceptible to cold plasma treatment, followed with TEN and AOH, respectively. There are three possible ways by which cold plasma treatment could exert such a reducing effect on *Alternaria* toxins content in wheat flour. These are heating, ultraviolet (UV) irradiation, and effects of plasma reactive species. As a non-thermal process, cold atmospheric plasma is designed to minimize heating of the samples. The temperature of SDBD treatments conducted in our experiment never exceeded 50 °C (the temperature recorded for the longest treatment period at the smallest distance of sample and plasma discharge). Thus, generally the temperature of the sample is well below the temperature required for thermal decomposition of *Alternaria* toxins. AOH and AME have melting points with decomposition at 350 °C and 267 °C, respectively, and undergo considerable reduction only after 20 min at 80 °C [43]. Hence, the contribution of heat is considered to have a negligible effect on *Alternaria* toxins degradation. The standard scores presented in Table 1 are calculated according to calculation explained in Section 4.10.3. The larger SS values are assigned to the more efficient toxins reduction (obtained by larger *t* and smaller *d* values).

As it can be observed from Figure 1, UV light was emitted due to the N_2_ species transition in the cold plasma generation process. Still, the emission power in cold plasma generation is not significant enough to be considered as effective for the degradation of mycotoxins, or in our study, *Alternaria* toxins [43]. Thus, toxin degradation should be attributed to the effects of plasma reactive species.

As earlier demonstrated by OES measurement, when SDBD plasma is excited, ROS and RNS were generated. Since these species accumulate over time of treatment, significant contribution to toxin degradation is possible. Based on the results of other studies, the authors believe that ROS, rather than RNS have a major role in toxin degradation under SDBD treatment. Justification for this claim can be found in the study [41] that reported much lower degradation efficiency of aflatoxin when nitrogen was used as a working medium for cold plasma generation instead of air. Moreover, while Shi et al. [41] were exploring the degradation mechanism, they could not find nitrogen moieties in the degradation products of aflatoxin. Holding to these findings by analogy, the authors expect that ROS are primarily responsible for *Alternaria* toxin degradation, but do not exclude the possibility of RNS contribution. One of the major ROS generated by cold atmospheric plasma is ozone O_3_ [44]. Ozone effects on mycotoxins degradation are known [45,46]. Several other ROS, atomic oxygen (O), the hydroxyl radical (OH•), and hydrogen peroxide (H_2_O_2_), accompany ozone in the generation of cold atmospheric plasma [26,39,44].

In addition, it was examined whether there is a correlation between process responses. The positive and highly significant correlations between AOH, AME, TEN and T can be observed in Table 2. Also, the negative, highly significant correlations between M and AOH, AME, TEN and T were obtained in Table 2.

**Figure 3:**
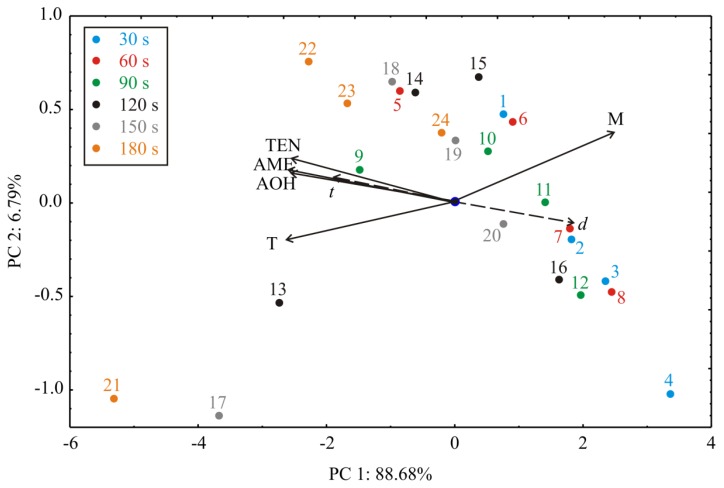
Principal Component Analysis (PCA) ordination of variables based on component correlations. *d*—distance of the cold plasma source to the sample; *t*—time range; AOH—reduction of alternariol; AME—reduction of alternariol monomethyl ether; TEN—reduction of tentoxin; T—temperature of wheat flour sample after treatment; M—moisture of wheat flour sample after treatment.

To characterize and differentiate among the observed samples pattern recognition techniques Principal Component Analysis–PCA were applied to the experimental data (used as descriptors). The points shown in the PCA graphics, which are geometrically close to each other, indicate the similarity of patterns that represent these points. The orientation of the vector describing the variable in factor space indicates an increasing trend of these variables, and the length of the vector is proportional to the square of the correlation values between the fitting value for the variable and the variable itself. The angles between corresponding variables indicate the degree of their correlations (small angles corresponding to high correlations). The PCA of the presented data explained that the first two components accounted for 95.47% of the total variance (88.68% and 6.79%, respectively) in the five variables system. Considering the map of the PCA performed on the data, AOH (which contributed 19.5% of total variance, based on correlations), AME (20.9%), TEN (19.7%) and T (21.2%) exhibited negative scores according to first principal component, whereas M (18.7%) showed a positive score value according to the first principal component (PC1) (Figure 3). The positive contribution to the second principal component (PC2) calculation was observed for: AME (10.9% of total variance, based on correlations), TEN (18.7%) and M (48.8%), while negative scores on the second principal component calculation was observed for T (13.3%).

The influence of processing parameters can be observed in Figure 3. According to the PCA results, the best results in reduction of AOH, AME and TEN were observed at 6 mm plasma source distance when the temperature of wheat flour sample after treatment was higher (this conclusion coincide with the SS results (Table 1.)) compared to other treatments. The processing time vector is oriented in the same direction as the AOH, AME and TEN vectors in the PCA graph, which means that time is positively correlated to the reduction of the AOH, AME and TEN, while the treatment variable is negatively correlated to the reduction of the AOH, AME and TEN. Groups of samples with the same processing time are coded using different colors, and it is evident that better results in the reduction of the AOH, AME and TEN were gained for longer processing time (orange color). The group of samples being treated for the same processing time differs due to the distance from the plasma source (treatment); if the distance to plasma source is shorter, better results in AOH, AME and TEN reduction is gained.

Further, ANOVA was conducted for obtained Second order polynomial (SOP) models, and output variables were tested against the impact of input variables (Table 3).

ANOVA analysis revealed that the linear terms of *d* and *t* considerably influenced the forming of SOP models for AOH, AME, TEN, T and M calculations, statistically significant at *p* < 0.05 level. The quadratic term of *t* was influential for the AOH reduction model, while the quadratic term of *d* was influential for the T and M SOP models. The interchange term *d* × *t* was influential for the SOP models of T and M prediction, statistically significant at *p* < 0.05 level. The coefficient of determination (*r*^2^) for the SOP models was rather good (Table 3). According to results presented in Table 3, the higher *r*^2^ values were attributed to SOP models in which the nonlinear terms were less effective and the linear terms of *d* and *t* were more pronounced.

Since the OES measurement performed in the present study can provide only qualitative information about reactive species, it is unclear which specific reactive species play the major role in *Alternaria* toxin degradation. The most likely scenario is degradation through the synergy of all ROS since they coexist during the treatment and are interconvertible. Besides the conditions already stated, degradation depends on relative humidity, as in humid air higher concentrations of OH• and H_2_O_2_ are achievable. During the samples treatment, the air was at an intermediate humidity level (40%). However, water evaporated from wheat flour samples into the surrounding air of cold plasma, which would have certainly resulted in an increase of OH• and H_2_O_2_ reactive species (Table 1). Therefore, it is expected that the degradation of *Alternaria* toxins was through combined effects of the following oxidative species: OH•, H_2_O_2_, and O_3_.

Furthermore, the quality of the model fit was tested in Table 4. The higher *r*^2^ values, and the lower χ^2^, MBE, RMSE and MPE values show the better fit to the experimental results [47]. The residual analysis of the developed model was also performed. Skewness measures the deviation of the distribution from normal symmetry. If the skewness is clearly different from zero, then the distribution is asymmetrical, while normal distributions are perfectly symmetrical. Kurtosis measures the “peakedness” of a distribution. If the Kurtosis is clearly different than zero, then the distribution is either flatter or more peaked than normal; the Kurtosis of the normal distribution is zero. The average and the standard deviation (SD) and the variance of residuals have also been analysed and shown in Table 4.

The residual analysis showed that the mean of residuals were equal to zero, and the standard deviation was between 0.140 and 3.939. These results showed a good approximation to a normal distribution around zero with a probability of 95% (2 × SD), which means a good generalization ability of the developed model for the range of observed experimental data.

Increasing the time of treatment increased the plasma degradation efficacy of toxins for all studied toxins in this study. Efficacy for treatment with 6 mm sample distance ranged from 21.5 %, 44.6% and 30.6% for 30 s plasma exposure to 60.7%, 73.8% and 54.5 % for AOH, AME and TEN respectively, for plasma exposure of 180 s. Observed reductions of *Alternaria* toxins can be explained with the increase of reactive species during longer treatment times. The increase in toxins degradation with time after 90 s is smaller than what one would expect based only on the first 60 s of sample exposure to cold plasma. We partly attribute this lower-than-expected increase in toxin degradation to the possibility that after the toxins are degraded in the surface layer of flour, degradation slows down due to smaller toxins availability in deeper layers. This is a specific wheat flour matrix feature, as samples were in powdered form, treatment time was probably spent on the diffusion of plasma reactive species into sample volume.

The SDBD expressed the filamentary nature of discharge across the electrode surface without directly making contact with samples placed below the electrode. This geometry certainly has an influence on the reactive species fluxes to the sample surfaces and on the effects of ROS and RNS. However, the remote production of ROS and RNS is significantly mediated by the diffusion time to the sample surface during the treatment. In all cases, degradation efficiency increases with increasing treatment time. As it can be seen from Table 1, degradation efficiency is greater for all toxins after an exposure time of 180 s at 51 mm than it is after an exposure time of 30 s and 6 mm distance from discharge. The extent of *Alternaria* toxin reduction might be dependent on the structure of mycotoxins in the first place, and then on their molecular mass. For example, AME with a molecular mass of 272.2 Da showed a greater reduction compared to AOH with a mass of 258.2 Da under the treatments studied. The reduction extent might be affected by their structure since these two mycotoxins share an identical structure with only one different group. AME is a benzochromenone that is AOH in which the hydroxy group at position 9 has been converted to the corresponding methyl ether. On the other hand, TEN is a natural cyclic tetrapeptide with molecular mass of 414.4 Da and exhibited higher reduction extent under almost all treatments studied compared to AOH. Our findings would be in accordance with the earlier results of other authors (summarized by ten Boch et al. [27]) that showed that the degradation of mycotoxins treated with atmospheric cold plasma did not correlate with a molecular mass. A hypothesis reported by other researchers suggests that mycotoxins with longer aliphatic chains are more sensitive to the influence of cold atmospheric plasma relative to mycotoxins with structures of condensed rings and aliphatic chains and mycotoxins with a compact structure of condensed aromatic rings [27]. Further, Standard Score Analysis (SS) of the five response variables was accomplished in order to find the processing variables (processing time and distance), that give optimal values of response variables. The “higher the better” or the “lower the better” criteria have been used according to the sign in “Polarity” raw in Table 1.

The standard score is the average of the five normal scores sum. Each response variable (the reduction of AOH, AME and TEN, wheat flour temperature and moisture content) has equal weight, when calculating the SS. The maximum of SS represents the optimal parameters for processing parameters, and also the optimum for response variables. SS analysis showed that the best results were obtained with treatment performed at 6 mm, during 180 s (SS was equal to 0.800, reduction of AOH was 60.6%, reduction of AME and TEN were 73.8% and 54.5%, respectively, while the obtained wheat flour temperature was 50 °C and the moisture was 12.3%). According to SS results, presented in Table 1, the satisfactory results for the observed toxins reductions were obtained at a shorter treatment distance (6 mm) and average processing time (120 and 150 s), at which the SS was 0.632 and 0.675. Using this set of process variables, relatively low reduction of AOH was performed. The different approach in optimization could be observed with average treatment distance (21 and 36 mm) and larger processing time (180 s), where gained SS reached 0.664 and 0.627. This set of variables lead to relatively low temperature of the wheat flour, but it also gained relatively lower reduction of all studied toxins.

### 2.3. Experimental Verification of the Mathematical Models

In order to test the accuracy of the developed mathematical models, the experimental verification of the model was performed. For the verification of models, the two previously untested values of distance of the cold plasma source to the sample, and the two time range values, within the tested range of values defined in Table 1, were chosen. The optimal process parameters (Trial 21 from Table 1, with *d* = 6 mm and *t* = 180 s) were also used in verification of the accuracy of the model. The experimental values of AOH, AME and TEN reduction were recorded, as well as the temperature and moisture of the observed material. The values of the response variables were also calculated. The results of the additional experiments and model calculated responses are presented in Table 5. According to the obtained results, only minor differences between the optimal experimental and predicted values for AOH, AME and TEN reduction, temperature and moisture content were observed, which means that the developed mathematical model could be used for prediction of the reduction of AOH, AME, TEN and also the temperature and moisture content of wheat flour.

## 3. Conclusions

It can be seen that similar results were obtained with all statistical analyses, pointing out that for Trial 21 the best score (SS was 0.800) was gained. The highest reduction of all three *Alternaria* toxins was achieved with treatment performed at 6 mm, during 180 s. Under these treatments, a reduction of 60.6%, 73.8% and 54.5%, for AOH, AME and TEN, respectively, was achieved. The results obtained in this study indicate that cold atmospheric plasma with SDBD excitation has the great potential for reduction of *Alternaria* toxin content. It can be stated that both investigated factors (time of exposure and distance from discharge) affect degradation efficiency of *Alternaria* toxins in wheat flour matrix. Authors attribute degradation effects to ROS of cold atmospheric plasma, and their synergies. Further, the authors do not exclude the effect of RNS completely. Thus, further research in this direction is needed. The ANOVA results revealed that the linear terms of *d* and *t* considerably influenced the forming of SOP models. The second order polynomial models showed good prediction capabilities (the coefficients of determination for the observed variables were between 0.927 and 0.961). On the basis of SOP models, the optimal treatment for toxin degradation was obtained at shorter treatment distance (6 mm) and longer time, with relatively low temperature of the wheat flour. In addition, future research should be related to the investigations of the effect of cold atmospheric plasma with SDBD excitation at optimal treatment conditions on the fate of *Alternaria* toxins by using naturally contaminated wheat milling products.

## 4. Materials and Methods

### 4.1. Material

For this study, white wheat flour was purchased at the market. The white wheat flour sample was analyzed before the spiking procedure, in order to confirm that it is a blank sample without any of the examined *Alternaria* toxins. In order to investigate the effect of atmospheric cold plasma on AOH, AME and TEN content, 10 g of white wheat flour was spiked with examined *Alternaria* toxins (100 μg kg^−1^ of each AOH, AME and TEN in flour).

### 4.2. Treating Spiked Samples with SDBD

**Figure 4:**
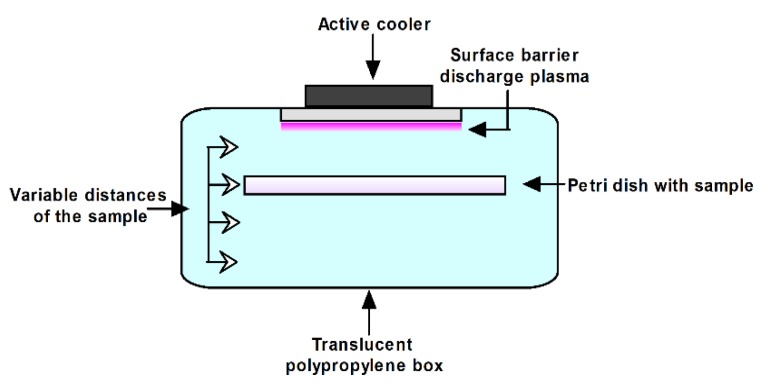
Schematic of experimental setup for SDBD treatment of *Alternaria* toxins in wheat flour.

The schematic of experimental set-up is shown in Figure 4. The plasma system consists of SDBD source, active cooler, translucent polypropylene box and a sample holder for a Petri dish of 100 mm diameter. The SDBD source has 9 stripe electrodes (~1mm width) placed in comb-like geometry on bottom sides of 2 mm ceramic dielectric plate (length 70 mm × width 40 mm). The distance between the stripes is 4 mm. These electrodes are placed along the plate length and connected to a high voltage (powered line from high-voltage (HV) transformer). The top of the dielectric surface is covered with a conductive layer which is connected to the ground line of the HV transformer. The plasma system was actively cooled by a cooler placed on the outside of the box cover on top of the SDBD. The electrical circuit of the plasma system consists of the SDBD source, commercial high-voltage transformer and variac regulator. The variac was powered through the standard electrical grid at the frequency of 50 Hz and it served as the regulator of HV given by the transformer. Stable plasma ignites in the surrounding air for the input voltage of 200 V given by the variac.

The box was sealed to prevent leakage of the plasma species that were generated. An amount of 10 g of spike white wheat flour was placed in Petri dish and subjected to SDBD treatment. Flour samples were treated for periods of 30 s, 60 s, 90 s, 120 s, 150 s and 180 s with variable distances from the SDBD plasma source, specifically 6 mm, 21 mm, 36 mm and 51 mm. The air in the box had a relative humidity (RH) of 45 ± 1% at room temperature. The parameters for the experimental set-up are shown in Table 6.

The temperature of the plasma was measured using an infrared thermometer (Fluke 64 MAX IR Thermometer, Everett, WA, USA). After treatment, the spiked samples were removed from the box, transferred to sealed bag and stored in freezer at 4 °C until mycotoxin analysis.

### 4.3. Optical Emission Spectroscopy

The optical emission spectroscopy (OES) of the surface barrier discharge in ambient air was captured in an empty polypropylene box, covering the near ultraviolet-visible region (270–850 nm). OES of the surface barrier discharge was acquired with a spectrometer (Shamrock 750, UK) with a detector (Andor DH734 ICCD camera, Belfast, UK) and optical fibers (Thorlabs, Newton, NJ, USA) with Ø200 µm core and collimating lens in UV and VIS range.

### 4.4. Electrical Measurements

Electrical measurements were performed by using a high-voltage probe (Tektronix, P6015A, Beaverton, OR, USA) and current probe (Agilent N2783B) which were placed in the electrical circuit close to the powered electrode. The waveform data were collected by using digital oscilloscope (Agilent, DSOX3014A, Waldbronn, Germany). Additionally, two multimeters were used to measure the voltage and current in the part of the circuit between the variac and high-voltage transformer. The root-mean-square values of voltage and current were used to determine voltage–current characteristics of the plasma system.

### 4.5. Moisture Content

Moisture content in white wheat flour samples before and after appleied treatments was determined using IM 9500 NIR instrument with the optional Flour Module (Perten Instruments, Hagersten, Sweden) and was expressed on the dry basis.

### 4.6. Sample Preparation

The modified method by Siegel et al. [48], described in detail in our previous studies [8,14], was used for sample preparation.

### 4.7. Instrumental Conditions

*Alternaria* toxins (AOH, AME and TEN) were quantified by high performance liquid chromatography coupled to tandem mass spectrometry (LC-MS/MS) using our previously published method [8] including the equipment and materials, but with some modifications. Namely, quantification of TEN (purity 99.2%) purchased from Sigma Aldrich (Seelze, Germany) was included in the method. TEN was quantified in negative ionization dynamic selected reactions monitoring mode, and was monitored at a determined retention time of ±1.5 min. Fragmentor voltage and collision energies were optimized during infusion of the pure standard of TEN (concentration of 5 µg mL^−1^), and the most abundant fragment ions were chosen for the selected reaction monitoring. The precursor ion for TEN was *m*/*z* 413.5, the fragmentor voltage for monitored product ions (*m*/*z* 141 and 271) was 170 V and the collision energies were 6 V and 3 V for *m*/*z* 141 and *m*/*z* 271, respectively. Retention time of TEN was 5.47 min.

### 4.8. Method Validation

The method was validated by an in-house quality control procedure following the guidelines of Commission Decision EC 657/2002 [49]. Method validation was performed in terms of matrix effects, linearity, trueness, precision, limit of detection (LOD) and limit of quantification (LOQ), by the same procedure, as were described in detail in our previous study [14].

The validation data of the analytical method for the determination of selected *Alternaria* toxins are given in Table 7. During the validation study, matrix-matched calibration (MMC) standards were used to compensate for the matrix effect, i.e., signal suppression or enhancement of the studied *Alternaria* toxins in the white wheat flour. AOH and TEN showed signal enhancement, while slight signal suppression was observed for AME.

The method exhibited good linearity, with correlation coefficients (*r*^2^) above 0.9924.

Trueness was evaluated through recovery studies. The overall method recoveries (*R_A_*) and the sample preparation recoveries (*R_E_*) for target analytes were calculated as were described in detail in our previous study [14]. It can be seen that the *R_A_* and the *R_E_* for all target analytes were above 70%, with the exception of *R_A_* for AME.

Precision for white wheat flour, expressed as the repeatability and within-laboratory reproducibility (Table 8), gave RSD values within the range of 3.4–11.2% and 6.1–11.9%, respectively, fulfilling the criteria of RSD ≤20% and indicating a good precision of the developed method.

### 4.9. Alternaria Toxins Determination

*Alternaria* toxins were quantified by external matrix-matched calibration procedure in order to eliminate the effect of matrix. Matrix-matched calibration curves were constructed in the concentration range from LOD to 100 µg kg^−1^ for AOH, AME and TEN, respectively. Linearity testing gave values of correlation coefficients (*r*^2^) above 0.9924 in all the investigated ranges. The obtained results were corrected for sample preparation recovery (*R_E_*), and were expressed on a dry matter basis. All samples were prepared and analyzed in triplicates. The reduction of AOH, AME and TEN were calculated as:

Reduction of *Alternaria* toxin (%) = 100 − (C_x_ × 100/C_0_) (1)

where C_x_ is the concentration of *Alternaria* toxins (AOH, AME and TEN) in the wheat flour sample after tretment; C_0_ is the initial concentration of *Alternaria* toxins (AOH, AME and TEN) in spiked wheat flour sample before tretment.

### 4.10. Statistical Analysis

The applied experimental design corresponded to a 4 × 6 Latin square design with two factors (Treat. – 4 levels and Time – 6 levels). The collected data were presented using descriptive statistics tables. The analysis and mathematical modelling was performed using STATISTICA 13.3 (V13.3; StatSoft, Inc.: Tulsa, OK, USA, 2018) [50].

#### 4.10.1. SOP Models

According to general recommendations, prior to artificial neural network (ANN) modelling, five SOP models were developed. SOP models were used for the modelling, rather than first order polynomials, due to the complexity of the data, and the pronounced nonlinear dependence between variables. Furthermore, ANOVA was performed, in order to explore the effects of the input variables over the outputs, as well as to justify the later use of the ANN model by the coefficient of determination (*r*^2^).

The SOP model was used for estimation of the main effect of the process variables on responses. The variables used for modelling were the reduction of AOH, AME and TEN, whey temperature (Temp) and a moisture of whey (M). The SOP model was fitted to data collected from experimental measurements [51,52]:

(2) $$Y_{k}=\beta_{0}+\sum_{i=1}^{3}\beta_{i}X_{i}+\sum_{i=1}^{3}\beta_{ii}X_{i}^{2}+\sum_{i=1}^{2}\sum_{j=2}^{3}\beta_{ij}X_{i}\cdot X_{j},\;k=1–8$$

where: *β*_0_, *β*_i_, *β*_ii_, *β*_ij_, are constant regression coefficients for intercept, linear, quadratic and product term, respectively, *Y_k_* is the response variable, while *X_i_* and *X_j_* are independent variables. The significant terms in the model were found using ANOVA for each dependent variable.

#### 4.10.2. The Accuracy of the Models

The numerical verification of the developed models was tested using coefficient of determination (*r*^2^), reduced chi-square (*χ*^2^), mean bias error (MBE), root mean square error (RMSE) and mean percentage error (MPE). These commonly used parameters can be calculated as follows [47]:

(3) $$\chi^{2}=\frac{\sum_{i=1}^{N}{\left(x_{\exp,i}-x_{pre,i}\right)}^{2}}{N-n}$$

(4) $$RMSE={\left[\frac{1}{N}\cdot \sum_{i=1}^{N}{\left(x_{pre,i}-x_{\exp,i}\right)}^{2}\right]}^{1/2}$$

(5) $$MBE=\frac{1}{N}\cdot \sum_{i=1}^{N}\left(x_{pre,i}-x_{\exp,i}\right),MPE=\frac{100}{N}\cdot \sum_{i=1}^{N}\left(\frac{\left|x_{pre,i}-x_{\exp,i}\right|}{x_{\exp,i}}\right)$$

where *x_exp,i_* stand for the experimental values and *x_pre,i_* are the predicted values by calculating from the model for these measurements. N and n are the number of observations and constants, respectively.

#### 4.10.3. Standard Score Calculation

Normal scores were calculated for each variable, and were used for complex comparison of observed samples, regarding their technological and chemical properties of the samples listed in Table 1. The ranking procedure between different samples was performed based upon the ratio of raw data and extreme values for each applied assay [53], according to these equations:

(6) $${\bar{x}}_{i}=1-\frac{\underset{i}{\max}\;x_{i}-x_{i}}{\underset{i}{\max}\;x_{i}-\underset{i}{\min}\;x_{i}},\;\forall i$$

in case of “the higher, the better” criteria, or

(7) $${\bar{x}}_{i}=\frac{\underset{i}{\max}\;x_{i}-x_{i}}{\underset{i}{\max}\;x_{i}-\underset{i}{\min}\;x_{i}},\;\forall i$$

in case of “the lower, the better” criteria.

where *x_i_* represents the raw data.

## Figures and Tables

**Table 1:** The results of cold plasma treatments on reduction of *Alternaria* toxins content in wheat flour.

Trial	Input *d* [mm]	*t* [s]	Responses AOH (%)*	AME (%) *	TEN (%) *	T (°C)	M (%)	SS
1	6	30	21.5	44.6	30.6	29.0	14.1	0.420
2	21	30	17.0	37.0	14.4	27.0	14.1	0.327
3	36	30	8.9	32.2	13.7	25.7	14.1	0.287
4	51	30	2.4	22.3	3.6	24.0	14.1	0.200
5	6	60	29.1	61.3	37.6	33.2	13.8	0.539
6	21	60	21.8	49.7	22.7	27.9	14.1	0.418
7	36	60	12.3	40.5	16.2	26.9	14.1	0.332
8	51	60	3.0	32.9	15.4	25.9	14.1	0.275
9	6	90	32.9	61.5	38.5	37.4	13.6	0.546
10	21	90	24.2	51.4	23.2	30.0	14.0	0.430
11	36	90	12.6	41.8	22.7	28.7	14.1	0.350
12	51	90	6.3	35.2	18.5	27.5	14.0	0.306
13	6	120	33.0	65.0	48.1	41.6	13.0	0.632
14	21	120	27.5	55.9	40.7	32.0	13.8	0.534
15	36	120	16.4	48.8	39.7	30.5	14.1	0.443
16	51	120	7.7	38.2	20.7	29.1	14.0	0.319
17	6	150	35.0	69.2	50.2	45.9	12.6	0.675
18	21	150	30.5	56.4	44.1	34.1	13.8	0.544
19	36	150	16.9	49.7	40	32.2	13.9	0.458
20	51	150	9.4	47.4	29.6	30.8	13.9	0.394
21	6	180	60.7	73.8	54.5	50.0	12.3	0.800
22	21	180	47.9	62.7	48.6	36.0	13.5	0.664
23	36	180	41.1	57.3	46.4	34.0	13.5	0.627
24	51	180	28.6	52.7	31.8	32.5	13.9	0.476
Polarity			+	+	+	−	−	

*d*—distance of the cold plasma source to the sample; *t*—time range; AOH—reduction of alternariol; AME—reduction of alternariol monomethyl ether; TEN—reduction of tentoxin; T—temperature of wheat flour sample after treatment; M—moisture of wheat flour sample after treatment; SS—standard score. Polarity—negative sign is associated with “the lower the better” criteria, while positive sign is associated with “the higher the better” criteria, as explained in Section 4.10.3. * Values are calculated (see Section 4.9).

**Table 2:** The correlation matrix of the process responses, during the cold plasma treatments on wheat flour.

Responses	AME	TEN	T	M
AOH	0.902	0.848	0.844	−0.784
AME		0.933	0.901	−0.792
TEN			0.861	−0.751
T				−0.963

AOH—reduction of alternariol; AME—reduction of alternariol monomethyl ether; TEN—reduction of tentoxin; T—temperature of wheat flour sample after treatment; M—moisture of wheat flour sample after treatment. * All correlations are statistically significant at *p* < 0.01 level.

**Table 3:** ANOVA calculation of the process responses, during the cold plasma treatments on wheat flour (sum of squares are presented).

Term	df	AOH	AME	TEN	T	M
*d*	1	2296.355 *	1942.272 *	1573.696 *	370.657 *	1.704 *
*d* ^2^	1	2.385	17.783	1.845	73.150 *	0.570 *
*t*	1	1892.277 *	1675.572 *	2626.082 *	391.053 *	1.808 *
*t* ^2^	1	344.607 *	21.607	6.396	0.142	0.066
*d* × *t*	1	26.331	4.890	2.185	52.807 *	0.890 *
Error	18	356.801	148.727	321.540	41.188	0.451
*r* ^2^		0.927	0.961	0.929	0.956	0.918

*d*—distance of the cold plasma source to the sample; *t*—time range; AOH—reduction of alternariol; AME—reduction of alternariol monomethyl ether; TEN—reduction of tentoxin; T—temperature of wheat flour sample after treatment; M—moisture of wheat flour sample after treatment; df—degrees of freedom; *r*^2^—coefficient of determination. * Statistically significant at *p* < 0.05 level.

**Table 4:** Goodness of fit for developed models of the process responses, during the cold plasma treatments on wheat flour.

Responses	*χ* ^2^	RMSE	MBE	MPE	*r* ^2^	Skew	Kurt	Mean	StDev	Var
AOH	18.779	3.856	0.000	19.474	0.927	−0.756	−0.179	0.000	3.939	15.513
AME	7.828	2.489	0.000	4.427	0.961	0.025	−0.072	0.000	2.543	6.466
TEN	16.923	3.660	0.000	11.794	0.929	−0.038	0.681	0.000	3.739	13.980
T	2.168	1.310	0.000	3.427	0.956	−0.579	−0.210	0.000	1.338	1.791
M	0.024	0.137	0.000	0.816	0.918	0.515	−0.177	0.000	0.140	0.020

AOH—reduction of alternariol; AME—reduction of alternariol monomethyl ether; TEN—reduction of tentoxin; T—temperature of wheat flour sample after treatment; M—moisture of wheat flour sample after treatment; *χ*^2^—reduced chi-square; RMSE—root mean square error; MBE—mean bias error; MPE—mean percentage error; *r*^2^—coefficient of determination; Skew—skeweness; Kurt—kurtoisis; Mean—mean values of residuals; StDev—standard deviation of residuals; Var—variance of residuals.

**Table 5:** Verification of the mathematical models of the process responses, during the cold plasma treatments on wheat flour.

Verification Trial	*d* (mm)	*t* (s)	AOH (%) *	AME (%) *	TEN (%) *	T (°C)	M (%)
Model	40	45	8.370	32.179	12.858	24.809	14.200
Exp.	40	45	8.498	31.980	12.754	24.767	14.249
Model	15	165	43.825	65.288	49.448	40.717	13.098
Exp.	15	165	44.408	64.022	50.256	40.852	13.027
Optimal	6	180	55.708	72.484	56.482	48.106	12.409

*d*—distance of the cold plasma source to the sample; *t*—time range; AOH—reduction of alternariol; AME—reduction of alternariol monomethyl ether; TEN—reduction of tentoxin; T—temperature of wheat flour sample after treatment; M—moisture of wheat flour sample after treatment. * Values are calculated (see Section 4.9).

**Table 6:** Parameters of the SDBD.

Parameter *	Value
Variac voltage/frequency	200 V/50 Hz
Applied RMS voltage/current	2340 V/0.094 A
Sample weight	10 g
Compartment box size (L × W × H)	195 mm × 125 mm × 65 mm
Gas temperature	≈40 °C

* L: length, W: width, H: height.

**Table 7:** Recovery data of the employed analytical method based on solvent (*R_A_*) and matrix-matched (*R_E_*) calibration curves and matrix effect (*SSE*)**.**

Analytes	Concentration Range (µg kg^−1^)	Overall Method Recovery *R_A_* (%) *	Sample Preparation Recovery *R_E_* (%) **	Matrix Effect *SSE* (%) ***	LOD/LOQ (µg kg^−1^)
AOH	2.5–100	90.4	80.4	112.5	0.75/2.5
AME	2.5–100	68.5	83.0	82.5	0.3/0.9
TEN	2.5–100	90.4	74.1	122.0	0.5/1.5

AOH—alternariol; AME—alternariol monomethyl ether; TEN—tentoxin; * Calculated by (slope of spiked sample-prepared curve/slope of solvent calibration curve); ** Calculated by (slope of spiked sample-prepared curve/slope of matrix-matched calibration curve); *** Calculated by (slope of matrix-matched calibration curve/slope of solvent calibration curve).

**Table 8:** Precision data of the selected *Alternaria* toxins.

Analytes	Spiking Level (µg kg^−1^)	Repeatability (*n* = 6) RSD (%)	Within-Laboratory Reproducibility (n = 3 × 6) RSDs (%)
AOH	25	11.2	11.8
	50	7.4	8.8
	100	6.0	7.7
AME	25	11.0	11.5
	50	7.0	7.2
	100	3.1	6.1
TEN	25	10.1	11.9
	50	5.3	9.8
	100	3.4	6.2

AOH—alternariol; AME—alternariol monomethyl ether; TEN—tentoxin; RSD (%)—relative standard deviation of 6 replicates at three concentration levels using the spiked white wheat flour and the matrix-matched calibration (MMC) curve; RSDs (%)—relative standard deviation of 6 replicates at three concentration levels using the spiked white wheat flour and the MMC curve, over the course of three days, using the same instrument and by the same operators.
